# RlmCD-mediated U747 methylation promotes efficient G748 methylation by methyltransferase RlmA^II^ in 23S rRNA in *Streptococcus pneumoniae*; interplay between two rRNA methylations responsible for telithromycin susceptibility

**DOI:** 10.1093/nar/gkv609

**Published:** 2015-10-10

**Authors:** Tatsuma Shoji, Akiko Takaya, Yoshiharu Sato, Satoshi Kimura, Tsutomu Suzuki, Tomoko Yamamoto

**Affiliations:** 1 Department of Microbiology and Molecular Genetics, Graduate School of Pharmaceutical Sciences, Chiba University, 1-8-1, Inohana, Chuo-ku, Chiba 260-8675, Japan; 2 Department of Chemistry and Biotechnology, Graduate School of Engineering, University of Tokyo, 7-3-1 Hongo, Bunkyo-ku, Tokyo 113-8656, Japan; 3 Division of Clinical Research, Medical Mycology Research Center, Chiba University, 1-8-1 Inohana, Chuo-ku, Chiba 260-8673, Japan

## Abstract

Adenine at position 752 in a loop of helix 35 from positions 745 to 752 in domain II of 23S rRNA is involved in binding to the ribosome of telithromycin (TEL), a member of ketolides. Methylation of guanine at position 748 by the intrinsic methyltransferase RlmA^II^ enhances binding of telithromycin (TEL) to A752 in *Streptococcus pneumoniae*. We have found that another intrinsic methylation of the adjacent uridine at position 747 enhances G748 methylation by RlmA^II^, rendering TEL susceptibility. U747 and another nucleotide, U1939, were methylated by the dual-specific methyltransferase RlmCD encoded by *SP_1029* in *S. pneumoniae*. Inactivation of RlmCD reduced N1-methylated level of G748 by RlmA^II^ *in vivo*, leading to TEL resistance when the nucleotide A2058, located in domain V of 23S rRNA, was dimethylated by the dimethyltransferase Erm(B). *In vitro* methylation of rRNA showed that RlmA^II^ activity was significantly enhanced by RlmCD-mediated pre-methylation of 23S rRNA. These results suggest that RlmCD-mediated U747 methylation promotes efficient G748 methylation by RlmA^II^, thereby facilitating TEL binding to the ribosome.

## INTRODUCTION

In prokaryotic protein synthesis, amino acids are polymerized into a polypeptide chain in the peptidyltransferase center (PTC) located in the 50S ribosomal subunit of the 70S ribosome. Newly synthesized polypeptides extrude through the nascent peptide exit tunnel (NPET), starting at the PTC and spanning the body of the 50S subunit, finally leaving the ribosome (1). The PTC consists of nucleotides from the central loop of domain V in 23S rRNA (2). A loop of helix 35 from domain II in 23S rRNA also lies directly adjacent to the PTC (3). Macrolides and ketolides, antibiotics that prevent protein synthesis on ribosomes, bind the upper chamber of the NPET between the PTC and the constriction formed by ribosomal proteins, L4 and L22, where they contact nucleotide A2058 located in the central loop of domain V (1). On binding, they hinder the passage of newly synthesized polypeptides through the NPET. Thus, the nucleotide changes and the altered modifications around PTC affect the antimicrobial activity of macrolides and ketolides.

**Figure 1.**
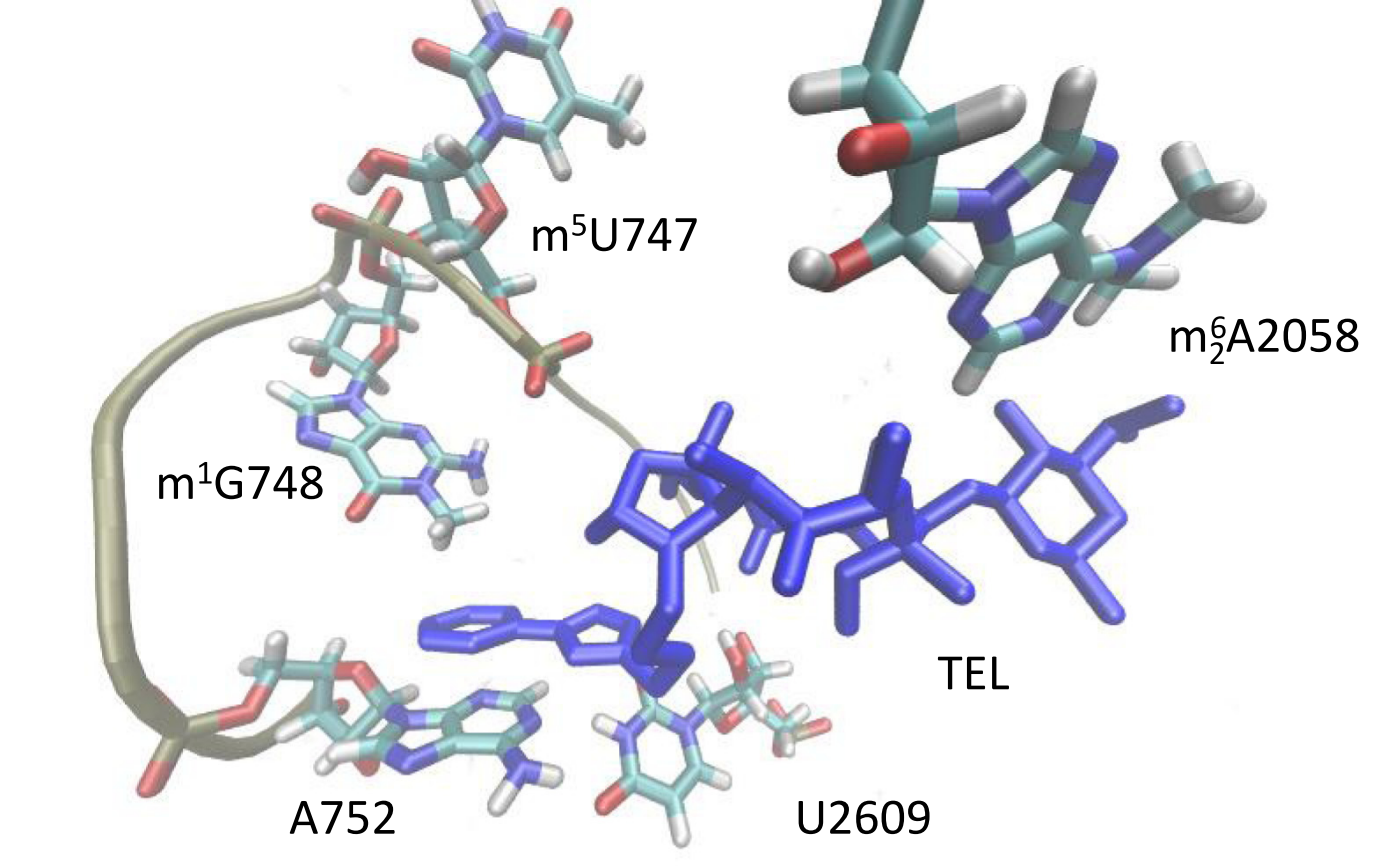
Structure of telithromycin (TEL) binding to domains II and V of 23S rRNA in 50S ribosomal subunit. TEL molecule is shown by a stick model. The loop of helix 35 (positions 745 to 752 in domain II) is represented by a tube model. The principal nucleotides (U747 and G748 in domain II, and A2058 and U2609 in domain V) are shown. U747, G748 and A2058 are shown in methylated models.

The most common mechanism of resistance to macrolides has been Erm methyltransferase, which is often acquired by bacteria through an exogenous gene. Erm mono- or di-methylates the N6 position of A2058 in domain V (4), and this prevents interaction with macrolides (5). In addition, the intrinsic methylation of N1 position of nucleotide G748 by methyltransferase RlmA^II^, encoded by *tlrB* gene on genome in some gram-positive bacteria, contributes to the resistance of tylosin, one of the 16-membered ring macrolides (6–8). G748 is located in the loop of helix 35 in domain II, close to the NPET constriction (1). The C_14_-linked mycinose of tylosin interacts with 2 nt, G748 and A752, in helix 35 (9). However, inactivation of RlmA^II^ results in increased resistance to telithromycin (TEL) when A2058 is dimethylated by Erm(B)-encoded dimethyltransferase in *Streptococcus pneumoniae*, as already deduced (10). TEL, a semi-synthetic derivative of the 14-membered macrolide, erythromycin A, is the first ketolide approved for clinical use. TEL is highly effective against *S. pneumoniae* isolates, causing community-acquired respiratory tract disease, including macrolide-resistant strains that have increased worldwide (11,12). TEL also interacts at nucleotide A752 through a synthetic alkyl-aryl substituent extending from C_11_ and C_12_ positions of the macrolide ring (Figure 1) (3). In *Escherichia coli*, the alkyl-aryl arm stacks on nucleotides A752 and U2609 to form a base-pair that bridges domains II and V in 23S rRNA (3). Molecular modeling shows that the methyl group of G748 pushes the alkyl-aryl arm of TEL toward the aromatic rings of A752 in the helix 35 loop, stabilizing the binding of TEL to the *S. pneumoniae* ribosome, even after dimethylation of A2058 (10). These results suggest that the individual modifications of nucleotides around the PTC alter the binding efficiency of macrolides and ketolides to the ribosome.

In *E. coli* 23S rRNA, 14 nt methylated by intrinsic methyltransferases are clustered around the PTC and the NPET (13). Most of the 23S rRNA methyltransferases seem to act at the early stage in ribosome assembly since they can only recognize the naked rRNA as a substrate *in vitro*. Although some modifications have been associated with maintenance of cellular health against the environmental stresses, including antibiotic attack, the roles of many modifications still remain unclear (13). In *E. coli*, nucleotide U747 located in the helix 35 loop is methylated at the C5 position by methyltransferase, RlmC, but function of the RlmC-methylation cannot be deduced since the disruption of *rlmC* leads to no significant change in phenotype (14).

To understand the function of methylation at U747, we examined the involvement of the methyl group at U747 in the hyper-susceptibility of *S. pneumoniae* to TEL because the methyl group at G748 next to U747 contributes to the TEL sensitivity. We initially searched for an RlmC homologue that modifies U747, finally identifying *S. pneumoniae* RlmCD as the enzyme mediating m^5^U747 and m^5^U1939 formation. Subsequent experimental analyses showed that RlmCD is required to enhance methylation of G748 by RlmA^II^, leading to TEL-susceptibility. RlmA^II^ prefers to recognize RlmCD-methylated 23S rRNA than the unmethylated 23S rRNA from RlmCD-disrupted strains *in vitro*. Thus we hypothesize that RlmCD-mediated U747 methylation promotes efficient G748 methylation by RlmA^II^, facilitating the binding of TEL to the ribosome.

## MATERIALS AND METHODS

### Bacterial strains, plasmids and media

Bacterial strains and plasmids are shown in Supplementary Tables S1 and S2, respectively. *S. pneumoniae* strain S1 with reduced TEL susceptibility (MIC, 2 μg/ml) was clinically isolated in Japan (10). Pneumococci were routinely cultured at 37°C and 5% CO_2_ in air in a brain-heart infusion with 0.5% yeast extract (BHI-Y) broth and BHI-Y agar, supplemented with 5% horse blood. *E. coli* was grown in L broth (1% Bact-tryptone, 0.5% Bact yeast extract, 0.5% sodium chloride, pH 7.4) and L agar. When necessary, the medium was supplemented with kanamycin (25–500 μg/ml), erythromycin (25 μg/ml), spectinomycin (100 μg/ml) and ampicillin (25 μg/ml).

### Transformation

Synthetic competence-stimulating peptide (CSP) 1 or 2 and the method of Iannelli and Pozzi (15) were used to transform *S. pneumoniae* S1 or TIGR4 into transformation-competent states, respectively.

### Antimicrobial susceptibility testing

Susceptibility to antibiotics was determined by the serial two-fold dilution method, using Mueller-Hinton agar plates supplemented with 5% lysed horse blood. Susceptibility or resistance of pneumococci to TEL was assessed as recommended by the Clinical and Laboratory Standards Institute (16).

### Construction of *rlmCD* and *SP_1901* disruption mutants

Disruption of *rlmCD* gene was constructed as follows. The fragment including a part of *rlmCD* (*SP_1029*) gene was amplified from chromosome of TIGR4 by colony direct polymerase chain reaction (PCR), using forward (5′-TTCCGTAAGAATTCGCATAACCTC-3′) and reverse primers (5′-CTGTTCGCATGCCAAGTAGGAATG-3′). The PCR product was digested with EcoRI-SphI and the fragment cloned into pUC18. The resultant plasmid, pTKY1157, was cleaved with AccI. The overhanging ends were blunted with T4 DNA polymerase and ligated to the fragment containing the spectinomycin resistance gene (Sp), generated from pTKY862 (17) after digestion with BamHI. This was followed by blunting with T4 DNA polymerase or the fragment containing the erythromycin resistance gene (Em), amplified from pAM225 (18) by PCR using forward (5′-GCGGATATCAGTTATGGAAATAAGACTTAG-3′) and reverse primers (5′-GCGGATATCTAGCTCCTTGGAAGCTGTCAG-3′), followed by digestion with EcoRV. The resultant plasmids, pTKY1174 and pTKY1205, were used to replace Δ*rlmCD*::Sp and Δ*rlmCD*::Em in *S. pneumoniae*, respectively.

Disruption of the *SP_1901* gene was constructed as follows: the fragment including a part of *SP_1901* gene was amplified from chromosome of TIGR4 by colony direct PCR, using forward (5′-TCGTCGAATTCTTGGAATGCAGGAAC-3′) and reverse primers (5′-GTTCAAGCTTTATGTGGGAACATATCGAC-3′). The PCR product was digested with EcoRI–HindIII and the fragment cloned into pUC18. The resultant plasmid, pTKY1156, was cleaved with XhoI. The overhanging ends were blunted with T4 DNA polymerase and ligated to the Sp fragment. The resultant plasmid pTKY1161 was used to replace Δ*SP_1901*::Sp in *S. pneumoniae*. Double-crossover events in all the constructed mutants were assessed by PCR.

### Construction of plasmids

To construct plasmid pTKY1121 encoding *tlrB*, the gene was amplified from the chromosome of TIGR4 by PCR, using the primers rlmAII-XhoI-F (5′-CTGTACTCGAGTACGGCAAGGCGACG-3′) and rlmAII-ApaI-R (5′-GGTTTGGGGCCCTGTTCTTATGCGTTTTG-3′). The fragment generated was cleaved with *X*hoI at the 5′ end and *Apa*I at the 3′ end and cloned into vector pLZ12-Km2. To construct a plasmid pTKY1196 encoding *rlmCD, rlmCD* gene was amplified from the chromosome of TIGR4 by colony direct PCR, using SP_1029-XhoI-F (5′-CGCCTCGACTAAAGAAAGTAAGGG-3′) and SP_1029-ApaI-R (5′-GCTGGGCCCATACCAAGTAGGAATG-3′) primers. The fragment generated was cleaved with XhoI at the 5′ end and ApaI at the 3′ end, and cloned into vector pLZ12-Km2.

To construct a plasmid, pTKY1201, for purification of RlmA^II^ protein, *tlrB* gene was amplified from the chromosome of TIGR4 by colony direct PCR, using gst-rlmAII-BamHI-F (5′-TAAGGATCCAATACAAATCTCAAGCCC-3′) and gst-rlmAII-XhoI-R (5′-TGACTCGAGTTAGAATGCTTTCCCAACC-3′) primers. The fragment generated was cleaved with BamHI at the 5′ end and XhoI at the 3′ end and cloned into vector pGEX-6p-1.

### Purification of rRNA

*Streptococcus pneumoniae* cultures (2.4L) were grown to log-phase and the cells were collected. The pellet was ground in a large mortar with 2.5× the pellet size of alumina at 4°C. The alumina and broken cells were suspended in ∼6 ml of Buffer A (10 mM HEPES-KOH, pH 7.6, 16 mM magnesium acetate, 50 mM NH_4_Cl, 0.1 mM DTT, 1 mM EGTA). DNaseI (Sigma) was added at 10 μl per ml, the suspension incubated at 4°C for 1 h and centrifuged at 8000 *g* for 15 min. The supernatants were centrifuged at 30 000 *g* for 30 min, and two-thirds of the supernatants were centrifuged at 30 000 *g* for 30 min before three-fourths of the supernatants were centrifuged at 105 000 *g* for 2 h to sediment the ribosomes. This pellet was suspended in 500 μl Buffer A and rRNA was extracted with RNeasy Mini kit (Qiagen).

### rRNA mass spectrometry

rRNA (100 fmol of 23S rRNA and 100 fmol of 16S rRNA) was digested at 37°C for 30 min in a 10 μl reaction mixture containing 10mM ammonium acetate (pH 5.3) and 5 U/μl RNase T1 (Epicentre). Subsequently, an equal volume of 0.1M triethylamine-acetate (TEAA) (pH 7.0) was added to the reaction mixture for LC/MS. Analysis of RNA fragments by capillary liquid chromatography (LC) coupled with nano electrospray (ESI) LC/MS was carried out using a linear ion trap-orbitrap hybrid mass spectrometer (LTQ Orbitrap XL, Thermo Fisher Scientific). The nomenclatures for product ions of nucleic acids are those suggested in reference (19).

### Primer extension to detect the methylated G748 in 23S rRNA

The degree of methylation of each RNA was assayed by a primer extension method (10).

### Purification of RlmA^II^

*Escherichia coli* BL21(DE3) was transformed with the plasmid pTKY1201 and the resultant transformants were grown at 37°C to an A_600_ of 0.5 in L broth containing 100 μg/ml ampicillin. Adding IPTG to 1 mM induced RlmA^II^ methyltransferase expression as a fusion protein with glutathione *S*-transferase. After overnight incubation at 20°C, cells were pelleted and resuspended in a pH 7.3 buffer (150 mM NaCl, 16 mM Na_2_HPO_4_, 4 mM NaH_2_PO_4_) containing 0.2 mM phenylmethyl sulfonyl fluoride. Cells were sonicated on ice and centrifuged at 8000 *g* for 30 min at 4°C. The supernatant was added to a Gultathione Sepharose 4 Fast Flow (GE Healthcare) equilibrated with a pH 7.3 phosphate buffer. After washing with a pH 7.5 buffer (50 mM Tris–HCl, 150 mM NaCl, 1 mM EDTA and 1 mM DTT), the fusion protein was incubated with 32 units of PreScission protease (GE Healthcare) overnight at 4°C and RlmA^II^ was collected by gravity-flow. This fraction was run on gel chromatography (Superdex75 16/600; GE Healthcare) with 20 mM HEPES-NaOH buffer at pH 7.5 containing 100 mM NH_4_Cl, 10 mM MgCl_2_, 10% glycerol and 6 mM β-mercaptoethanol. To concentrate RlmA^II^, the fractions containing RlmA^II^ were loaded onto RESOURSE Q column (1 ml; GE Healthcare), equilibrated in buffer (20 mM HEPES-NaOH, pH 7.5, 10 mM MgCl_2_, 10% glycerol, 5 mM DTT) and eluted with buffer A containing a 0–1M NaCl linear gradient. The peak fraction was used as the purified RlmA^II^ protein.

### Generation of anti-RlmA^II^ antibody

A 1.8 ml portion of the peak fraction was used to immunize a rabbit to obtain antiserum against RlmA^II^. For purification, purified RlmA^II^ was added to a CNBr-activated Sepharose 4 Fast Flow equilibrated with buffer containing 20 mM HEPES-NaOH, pH 7.5, 10 mM MgCl_2_, 10% glycerol, 5 mM DTT and 300 mM NaCl, and incubated at room temperature for 4 h. After washing excess RlmA^II^ with coupling buffer (1M NaHCO_3_ pH 8.3, 500 mM NaCl), Sepharose was incubated with 0.1M Tris–HCl (pH 8.0) at 4°C for 2 h followed by washing in low and high pH buffer. Anti-RlmA^II^ antiserum was added to the Sepharose equilibrated with TBS-T (100 mM Tris, 150 mM NaCl, 0.1% Tween20), incubated at 4°C for 2 h, washed with TBS-T followed by buffer (5 mM glycine-HCl, pH 2.3, 0.5% Tween20, 100μg/ml BSA) containing 500, 600, 700, 800 or 900 mM NaCl. Anti-RlmA^II^ was eluted with buffer containing 3M NH_4_SCN, 150 mM KCl, 10 mM sodium phosphate at pH 6.0 and 100 μg/ml BSA, which was analyzed by immunoblotting analysis.

### Immunoblotting

*Streptococcus pneumoniae* culture was grown to stationary phase, collected, sonicated and separated on 11% sodium dodecyl sulphate-polyacrylamide gel electrophoresis gels. The proteins were transferred onto membrane (Hybond-P; GE Healthcare) and then incubated with anti-*S. pneumoniae* RlmA^II^ (1:750), followed by HRP-conjugated anti-rabbit immunoglobulin G. The enzyme was detected by Amersham™ ECL™ Prime Western Blotting Detection Reagent (GE Healthcare).

### *In vitro* methylation assay

The rRNA was dissolved in 200 μl buffer containing 20 mM HEPES-NaOH (pH 7.5), 100 mM NH_4_Cl, 10 mM MgCl_2_, 10% glycerol and 6 mM β-mercaptoethanol at a final concentration of 10 nM. RNAs were renatured by heating at 50°C for 5 min and 5 min at 37°C. *S*-Adenosylmethionine was added to 0.5 mM and incubated followed at 37°C for 5 min. RlmA^II^ was added to 10–2000 nM to start the methylation reaction that ran for 0–150 min at 37°C. Methylation reactions were stopped by extraction with phenol and chloroform, and the RNAs recovered by ethanol precipitation. Methylation at G748 was quantified by primer extension.

## RESULTS

### Identification of the methyltransferase RlmCD encoded by *SP_1029*

To identify a methyltransferase that modifies nucleotide U747 in the *S. pneumoniae* 23S rRNA, two *S. pneumoniae* orthologues, SP_1029 and SP_1901, were examined as candidates by a BLAST search of the *S. pneumoniae* TIGR4 genome, using the sequence of the *E. coli* m^5^U methyltransferase RlmC as the query. These putative *S. pneumoniae* methyltransferases are 33% identical (74% similar) in their amino acid sequences. Comparison with the *E. coli* methyltransferase showed that SP_1029 and SP_1901 have 23 and 24% identity (65 and 63% similarity) to RlmC, respectively (Supplementary Figure S1) from which we predicted that *SP_1029* and *SP_1901* are candidates for genes encoding enzymes that catalyze the m^5^U modification at U747 in the *S. pneumoniae* 23S rRNA.

**Figure 2.**
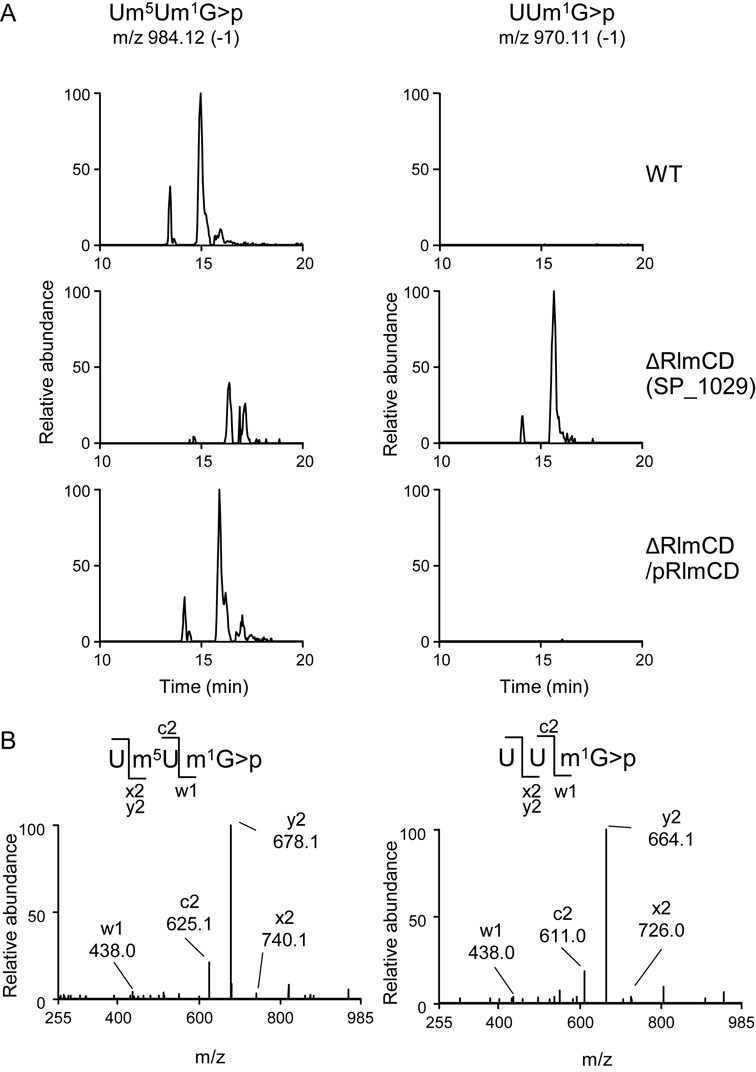
*rlmCD* (*SP*_*1029*) is responsible for m^5^U747 formation. (**A**) LC/MS analysis of RNaseT1 digest of rRNAs prepared from strains TIGR4 (WT; top row of the panels), Sp332 (ΔRlmCD; middle row) and Sp361 (ΔRlmCD/pRlmCD; bottom row). Left and right panels show mass chromatographic detection of singly-charged ions of 3-mer fragment m^5^U747 (*m/z* 984.12) and U747 (*m/z* 970.10), respectively. (**B**) Collision-induced dissociation (CID) spectra of RNA fragments bearing m^5^U747 (left panel) and U747 (right panel). The singly-charged ions of the 3-mer fragment carrying m^5^U747 (*m/z* 984.12) and U747 (*m/z* 970.11) were used as parent ions for CID.

To determine whether both candidate genes encode *S. pneumoniae* methyltransferase for modifying m^5^U747, we constructed *SP_102*9-disrupted and *SP_1901*-disrupted mutants by inserting the spectinomycin resistance gene *aad(9)* into each gene of strain TIGR4 and analyzed the m^5^U-methylation site in the resulting mutant cells, with wild-type cells as the control. The fragment, Um^5^Um^1^Gp, obtained from total RNAs digested with RNase T1 was analyzed by capillary LC coupled with nano ESI LC/MS system (Figure 2A). The fragment containing modified nucleosides could be detected as multiple-charged negative ions by monitoring the specific mass-to-charge ratio (*m/z*) value. In the MS spectra of the RNase T1-digests of RNA from the wild-type strain, the fragment produced peaks at *m/z* 984.12 as a single-charged negative ion with 3′-cyclic phosphate form (Figure 2A), indicating that U747 can be methylated in the *S. pneumoniae* 23S rRNA. The peak at *m/z* 984.12 was also present in the digests of RNA from *SP_1901*-disrupted cells (data not shown). In contrast, the methylated fragment from the rRNA of the *SP_1029*-disrupted cells was not detected, but a fragment was detected at *m/z* 970.1, the difference of 14 Da compared to the wild-type rRNA corresponding to the loss of one methyl group (Figure 2A). G748 in the fragment was also methylated by another methyltransferase RlmA^II^. To confirm the exact position of the modification by SP_1029, each fragment was analyzed by collision-induced dissociation (CID) in the MS/MS system (Figure 2B), which showed that the methyl group was removed from the second U corresponding to position 747 when *SP_1029* was disrupted. The loss of the mass of one methyl group due to *SP_1029*-disruption was complemented in strain Sp361 when the pTKY1196 plasmid, harboring *SP_1029* of strain S1, was provided (Figure 2A). The data indicate that the methyltransferase encoded by *SP_1029* methylates U747 in the *S. pneumoniae* 23S rRNA.

**Figure 3.**
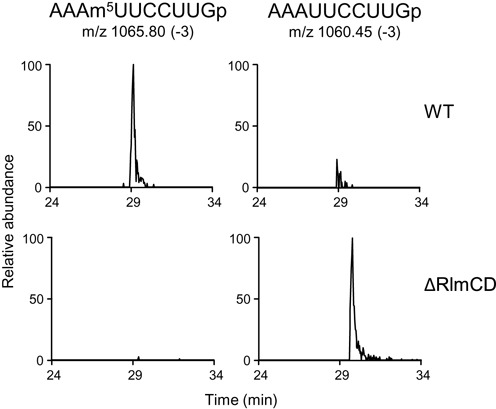
*rlmCD* (*SP*_*1029*) is also responsible for m^5^U1939 formation. LC/MS analysis of RNaseT1 digest of rRNAs prepared from TIGR4 (WT; upper row of panels) and Sp332 (ΔRlmCD; lower row). Left and right panels show mass chromatograms detecting triply-charged ions of 10-mer fragment bearing m^5^U1939 (*m/z* 1065.80) and U1939 (*m/z* 1060.45), respectively.

*Bacillus subtilis* YefA, to which SP_1029 shows 42% identity (81% similarity) in amino acid sequence (Supplementary Figure S1), methylates not only U747 but U1939 in domain IV of 23S rRNA (20). In *E. coli*, methylation at U1939 is catalyzed by another methyltransferase RlmD (14). To examine the involvement of SP_1029 as responsible for m^5^U1939 modification in *S. pneumoniae*, we also analyzed a 10-mer fragment containing U1939 in the mixture of RNA digested by RNase T1 (Figure 3). In the wild-type cells, a fragment including m^5^U1939 (AAAm^5^UUCCUUGp) was detected at *m/z* 1065.80 as the triple-charged negative ion. In the *SP_1029*-disrupted cells, a fragment lacking one methyl group (AAAUUCCUUGp) was detected at *m/z* 1060.45, indicating that SP_1029 can also catalyze methylation at U1939 as well as U747. Therefore, we conclude that *S. pneumoniae SP_1029* encodes the dual-specific methyltransferase RlmCD.

### RlmCD functioning is involved in the TEL sensitivity

To examine the contribution of RlmCD to the susceptibility of *S. pneumoniae* to TEL, we determined the TEL MIC of strain Sp338, which is a RlmCD-disrupted mutant harboring pTKY1041 encoding *erm*(B) of strain S1 (10) because wild-type and RlmCD-disrupted mutant of strain TIGR4 had high TEL susceptibility (MIC, <0.03 μg/ml) (Table 1). The *erm*(B) responsible for the constitutive dimethylation at the N6 position of A2058 in domain V of 23S rRNA was provided because dimethylation is necessary to reduce TEL susceptibility in strain TIGR4 (MIC, 2 μg/ml). Susceptibility testing of strain Sp338 showed that RlmCD disruption increased resistance to TEL (MIC, 8 μg/ml). The RlmCD-disrupted mutant Sp345 derived from strain S1, with reduced TEL susceptibility (MIC, 2 μg/ml), had increased resistance to TEL (MIC, 8 μg/ml) (Table 1). TEL resistance due to RlmCD disruption was complemented (MIC, 2 μg/ml) in strain Sp360 when the pTKY1196 plasmid carrying *rlmCD* of strain S1 was provided. These results suggest that RlmCD is functionally involved in TEL susceptibility in *erm*(B)-carrying *S. pneumoniae*.

**Table 1.** TEL-MICs of the ΔRlmCD mutants derived from *S. pneumoniae* TIGR4 and S1 strains

Original strain	*erm*(B)	Characteristics	Designation of mutants	MIC (μg/ml)^a^
TIGR4	none	RlmCD^+^		<0.03
		ΔRlmCD	Sp332	<0.03
	plasmid	RlmCD^+^	Sp224	2
		ΔRlmCD	Sp338	8
S1	chromosome	RlmCD^+^		2
		ΔRlmCD	Sp345	8
		ΔRlmCD/pRlmCD	Sp360	8
		ΔRlmCD/pRlmA^II^	Sp355	8

^a^Values for at least three independent experiments are given.

### RlmCD enhances the methylating level at G748 by RlmA^II^

**Figure 4.**
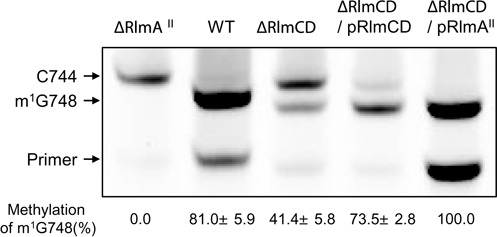
RlmCD promotes the methylation at G748 in 23S rRNA. rRNAs prepared from strains S1 (WT), Sp274 (ΔRlmA^II^), Sp345 (ΔRlmCD), Sp355 (ΔRlmCD/pRlmA^II^) and Sp360 (ΔRlmCD/pRlmCD) were sequenced by reverse transcriptase extension from bases 756 to 790. N1-methylation at G748 aborts extension. The band corresponding to methylation at G748 relative to the total band was quantified. Mean values and standard deviations from at least three independent experiments are given.

In the helix 35 loop that includes U747 of 23S rRNA, nucleotide A752 mediates the binding of TEL to the ribosome by interacting with the alkyl-aryl arm of TEL (3). We know that this interaction is stabilized by the methyl group of G748, which is catalyzed by the metyltransferase RlmA^II^, based on the molecular modeling (10). The loss of RlmA^II^ functioning therefore results in low TEL susceptibility but not affect the dimethylated level of A2058 by Erm(B). Similarly, primer extension analysis showed that RlmCD disruption did not affect the degree of dimethylation at A2058 by Erm(B) (data not shown). To examine the effect of RlmCD disruption on methylation at G748, basically modified by RlmA^II^, the level of N1-methylation at G748 in the 23S rRNA of RlmCD-disrupted mutant derived from strain S1 was determined by reverse transcriptase extension from bases 756 to 790 to pause at m^1^G748 (Figure 4). The top band in the strain S1 lane in Figure 4 corresponds to m^1^G748, whereas the top band in the RlmA^II^-deficient mutant Sp274 lane corresponds to C744 because the reverse transcriptase stops synthesizing the DNA strand when it reaches the cytosine at nucleotide 744 because ddGTP is in the mixture. Both bands can be seen in the RlmCD-disrupted mutant, Sp345 lane (Figure 4, lane 3). The density of the band corresponding to m^1^G748 was significantly lower than in wild-type cells. Quantification of the m^1^G748 band in RlmCD-disrupted mutant was 41.4 ± 5.8%, whereas in strain S1 it was 81.0 ± 5.9%. Reduced methylation was complemented when the pTKY1196 plasmid was present (Figure 4, lane 4; 73.5 ± 2.8%), similar results being obtained from the strain TIGR4 (Supplementary Figure S2). These results suggest that functional RlmCD promotes G748 methylation *in vivo*.

To determine the effect of a reduced level of m^1^G748 on TEL resistance in RlmCD-disrupted mutant, the pTKY1111 plasmid carrying RlmA^II^ gene of strain S1 was introduced to the RlmCD-disrupted mutant Sp345. G748 was completely methylated in the 23S rRNA from the resultant strain, Sp355 (Figure 4, lane 5). TEL susceptibility was also complemented (MIC, 2 μg/ml) in strain Sp355 (Table 1). We conclude that RlmCD functionally enhances the methylation of G748 by RlmA^II^, which is sufficient for TEL susceptibility in *S. pneumoniae*.

### RlmCD disruption does not influence the level of RlmA^II^

**Figure 5.**
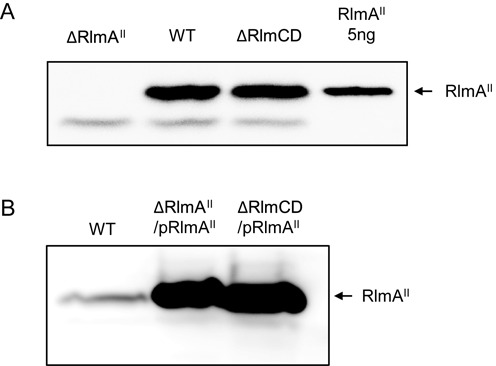
Effect of RlmCD-depletion on RlmA^II^. Whole cell lysates were prepared from strains TIGR4 (WT), Sp303 (ΔRlmA^II^) and Sp332 (ΔRlmCD) (**A**) and Sp322 (ΔRlmA^II^/pRlmA^II^) and Sp370 (ΔRlmCD/pRlmA^II^) (**B**) grown in BHIY broth to A_600_ of 1.0 at 37°C and 5% CO_2_ in air, and separated on SDS-11% polyacrylamide gels, with RlmA^II^ (5 ng) being the positive control. Proteins were immunostained with RlmA^II^ antibody.

RlmA^II^ is a unique enzyme for methylating the N1 position at G748 because m^1^G748 disappeared completely in the RlmA^II^-disrupted mutant (Supplementary Figure S2). To determine whether RlmCD disruption affects the level of RlmA^II^ protein, we initially constructed a plasmid encoding GST fused RlmA^II^ and then purified a native RlmA^II^ protein from the GST-fused RlmA^II^ protein digested with PreScission protease. The purified protein was used to immunize a rabbit. Immunoblotting analysis is shown of the levels of RlmA^II^ in the strains depicted in Figure 5. A band corresponding to 32 kDa in the wild-type cells was not present in the RlmA^II^-disrupted cells (Figure 5A). The lack of the protein in the RlmA^II^-disrupted mutant, Sp303, was complemented by adding the plasmid containing RlmA^II^, pTKY1111 (Figure 5B). These results indicate that the antibody specifically recognizes the level of RlmA^II^. In comparison RlmCD disruption did not influence the RlmA^II^ level (Figure 5A).

### RlmCD-modification of 23S rRNA enhances the RlmA^II^ activity

**Figure 6.**
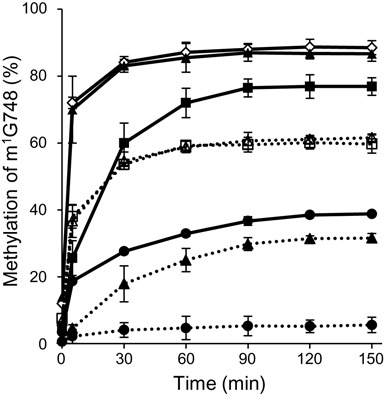
Prior methylation of 23S rRNA by RlmCD enhances m^1^G748 formation by RlmA^II^*in vitro*. rRNAs (10 nM) prepared from strains Sp303 (RlmCD modified-23S rRNA; solid lines) and Sp369 (RlmCD unmodified-23S rRNA; dot lines) were catalyzed by concentration of RlmA^II^ at 10 (closed circle), 20 (closed square), 100 (closed triangle), 200 (open diamond), 1000 (open triangle) and 2000 nM (open square). Methylation was stopped using phenol solution at the given times and the amount of m^1^G748 analyzed by reverse transcriptase extension. The ratio of m^1^G748 formation (%) was calculated from the band corresponding to m^1^G748 relative to the total band. Mean values and standard deviations from at least 3 independent experiments are given.

When the plasmid encoding RlmA^II^ was added in the RlmCD-disrupted mutant, the RlmA^II^ level was significantly increased compared with the wild-type cells (Figure 5B), which indicates that an excess of RlmA^II^ allows 23S rRNA to promote the methylation at G748 even in the absence of RlmCD (Figure 4). We therefore hypothesized that RlmCD-modification of 23S rRNA enhances RlmA^II^ recognition of G748 and to examine this possibility, the *in vitro* activity of the purified RlmA^II^ was analyzed using 23S rRNAs from the RlmA^II^-disrupted mutant Sp303 (RlmCD modified-23S rRNA) and the RlmA^II^ and RlmCD-double disrupted mutant Sp369 (RlmCD unmodified-23S rRNA) as substrates. RlmCD modified-23S rRNA (10 nM) was reacted with different concentrations of the purified RlmA^II^. Aliquots were mixed with phenol solution to stop methylation. The level of m^1^G748 was detected by primer extension analysis (Figure 6, Supplementary Figure S3). When RlmCD modified-23S rRNA was used, 10nM RlmA^II^ could methylate 17% of G748 after 5 min and 39% by 2 h (Figure 6, Supplementary Figure S3 upper panel). Furthermore, ∼87% of G748 could be methylated by reacting with 100 nM RlmA^II^. These results confirm that the purified RlmA^II^ retained G748 methylating activity. On the other hand, when RlmCD unmodified-23S rRNA was used, 10 nM RlmA^II^ could methylate only 7% of G748 even after 2 h (Figure 6, Supplementary Figure S3 lower panel). When 100 nM RlmA^II^ was reacted for 2 h, 31% of G748 was methylated, a yield almost the same as the level of methylation when RlmCD modified-23S rRNA was reacted with 10 nM RlmA^II^. When 1000 nM RlmA^II^ was used, the initial rate was restored to that of RlmCD modified-23S rRNA with 20 nM RlmA^II^, but only 62% of G748 attained the methylation level after 2 h. From these results, we suggest that the prior modification of 23S rRNA by RlmCD enhances RlmA^II^ methylating activity to G748.

## DISCUSSION

Uridine at 747 located within the loop of helix 35 is a highly conserved residue, as is the uridine at 1939 located within the 1942 loop in domain IV (21). In *E. coli*, the C5 positions of U747 and U1939 are methylated post-transcriptionally by RlmC and RlmD, respectively (14). In *B. subtilis*, YefA catalyzes C5-methylation of both uridines (20). We found that U747 and U1939 were methylated by RlmCD encoded by *SP_1029* in *S. pneumoniae* (Figures 2 and 3). In the minimalist bacterium, *Mycoplasma capricolum*, C5-methylation at U1939 is also catalyzed by the flavoprotein, RlmFO (22), which suggests that C5-methylations at U747 and U1939 are highly conserved modifications in the large subunit of bacterial ribosomes. However, the role of C5-methylations at U747 and U1939 in maintaining cell viability remains unclear, even using knockout studies of the C5-methyltransferase in *E. coli, B. subtilis* and *M. capricolum* (20,22–23). We found that RlmCD activity contributes to TEL sensitivity of Erm(B)-expressing *S. pneumoniae* (Table 1). The loop containing m^5^U1939 forms inter-subunit bridges with 16S rRNA (24) and this residue does not contribute to macrolide–ribosome interactions (24). On the other hand, the m^5^U747 is located near the TEL binding region. Although the binding of TEL to A2058 is weakened by dimethylation with Erm(B), the interaction of alkyl-aryl group of TEL with A752 located in helix 35 by π–π packing persists (3,10). The methyl group of N1 position at G748 also interacts with alkyl-aryl group through π-CH, stabilizing TEL-ribosome binding (10). Although U747 is also located in helix 35, the methyl group in the C5 position at U747 is located on the opposite site to the alkyl-aryl group of TEL (Figure 1). Therefore, the methyl group of U747 could not directly affect the interaction between the alkyl-aryl arm of TEL and A752.

The mechanism of TEL-ribosome stabilization by RlmCD involves its modifications enhancing N1-methylation of G748 catalyzed by RlmA^II^ (Figures 4 and 6). Without U747 methylation by RlmCD, full methylation at G748 could still be attained by increasing the amount of RlmA^II^ (Figures 5 and 6), which suggests that the methylated U747 or/and U1939 by RlmCD efficiently guides the 23S rRNA to sequential methylation by RlmA^II^. In the crystal structures of ribosomal particles, G748 and A2058 are ∼15 Å apart on opposite faces of NPET (24–27). RlmA^II^ could not access its target G748 within the 50S subunit and methylates G748 in the free 23S rRNA prior to assembly with ribosomal proteins (28,29). Therefore, RlmCD can recognize free 23S rRNA; in fact, *E. coli* RlmD can methylate U1939 in the free 23S rRNA (30,31). For RlmA^II^ recognition, the specific surface shape at the 3-way junction formed by helices 33, 34 and 35 is required, the essential recognition element being located in helix 35 (28,29). Therefore, the methyl group of U747 may contribute to the recognition of 23S rRNA by RlmA^II^. Lebars *et al*. (28) showed that the full-length 23S rRNAs from the *rlmA^I^*-defective strain of *E. coli* and *Streptococcus pyogenes*, which does not originally have the *rlmA^II^* gene, were better substrates than the streptococcal 76 nt RNA transcript containing helices 33, 34 and 35 from *S. pneumoniae* 23S rRNA. U747 in *E. coli* 23S rRNA is methylated by RlmC, but U747 in *S. pyogenes* 23S rRNA could also be methylated by the RlmCD homologue (Accession No. WP_032462573; 76% identity and 88% similarity to *S. pneumoniae* RlmCD). However, U747 in the 76 nt RNA transcribed by the *in vitro* transcription system was unmodified. Our finding is supported by the fact that unmodified U747 in the 76 nt RNA is due to reduced RlmA^II^ reactivity.

Like RlmA^II^, the activity of only a few methyltransferases is influenced by other modification of nucleotides. An example of cooperative rRNA modification is two methylations by a fused methyltransferase RlmKL (32). RlmKL consists of two methyltransferases, RlmK and RlmL responsible for 7-methylation at G2069 and N2-methylation at G2445, respectively. In the case of RlmKL, although methylation state did not affect the efficiency of the other methylation, the substrate binding of RlmK domain enhanced the methylation of RlmL through unwinding the duplex substrate. For another example, modification of rRNA by RlmH, the pseudouridine-specific methyltransferase, is necessary for the pre-modification of the target nucleotide U1915. U1915 is modified to pseudouridine by the pseudouridine synthase, RluD and is then methylated by RlmH (33). RlmH prefers pseudouridine to uridine as the methylation target (33,34). This pre-modification contributes to the substrate recognition of the methyltransferase. *In vitro* methylation of G748 by RlmA^II^ suggests that RlmCD-mediated U747 methylation also enhances RlmA^II^ recognition of 23S rRNA (Figure 6). RlmA^II^ recognition of 23S rRNA is dependent on a specific shape, the W-shaped RNA binding cleft in the RlmA dimer (35). The docking study of RlmA and 23S rRNA indicates that the rRNA-binding cleft corresponds to the rRNA structure containing helices 33, 34 and 35 (35). The angle between helices 33 and 35 is important in this interaction, and the methyl group of U747 may contribute to its maintenance. The docking study also shows that helix 35 is completely buried in this W-shaped cleft (35). Two SAM-binding pockets are located at the bottom of the cleft whereas only one base of the rRNA substrate is methylated by RlmA^II^. Therefore, G748 in helix 35 points precisely toward one SAM-binding pocket in the cleft. U747 is also located in the cleft and its methyl group may help the G748 positioning.

Although *S. pneumoniae* has another RlmCD homologue, SP_1901, this is not involved in methylation at U747 and U1939 (Figure 2, and data not shown). It has 47% identity (80% similarity) to YfjO, which is the unknown methyltransferase in *B. subtilis* (20). *Acholeplasma laidlawii* 23S rRNA has up to 6 m^5^U nucleotides, whereas *E. coli* 23S rRNA has 2 m^5^U nucleotides (36). *S. pneumoniae* 23S rRNA might have one or more uridine(s) methylated by SP_1901.

In conclusion, methylation of 23S rRNA by the dual-specific methyltransferase, RlmCD, guides the 23S rRNA efficiently to sequential methylation by the other methyltransferase, RlmA^II^, rendering *S. pneumoniae* TEL susceptible. The RlmA^II^-encoding gene, *tlrB*, was first identified as a tylosin resistance gene within the tylosin biosynthetic cluster from a tylosin-producing *Streptomyces fradiae* (37,38). In the actinomyces, U747 is conserved in helix 35. Our findings suggest that the activity of a methyltransferase-related antibiotic resistance is enhanced through the modification due to the intrinsic methyltransferase of other nucleotides of rRNA, leading to the antibiotic sensitivity.

## SUPPLEMENTARY DATA

Supplementary Data are available at NAR Online.

SUPPLEMENTARY DATA
