# Supplementary material for: RlmCD-mediated U747 methylation promotes efficient G748 methylation by methyltransferase RlmAII in 23S rRNA in Streptococcus pneumoniae; interplay between two rRNA methylations responsible for telithromycin susceptibility
Source: Nucleic Acids Res. 2015 Oct 10;43(18):8964–72. doi: 10.1093/nar/gkv609 (PMC4605293; doi:10.1093/nar/gkv609)
Supplement: SUPPLEMENTARY DATA [file supp_43_18_8964__index.html]

RlmCD-mediated U747 methylation promotes efficient G748 methylation by methyltransferase RlmAII in 23S rRNA in Streptococcus pneumoniae; interplay between two rRNA methylations responsible for telithromycin susceptibility — RlmCD-mediated U747 methylation promotes efficient G748 methylation by methyltransferase RlmAII in 23S rRNA in Streptococcus pneumoniae; interplay between two rRNA methylations responsible for telithromycin susceptibility — SUPPLEMENTARY DATA 

# RlmCD-mediated U747 methylation promotes efficient G748 methylation by methyltransferase RlmAII in 23S rRNA in *Streptococcus pneumoniae*; interplay between two rRNA methylations responsible for telithromycin susceptibility

## SUPPLEMENTARY DATA

- SUPPLEMENTARY DATA
