# Supplementary material for: RlmCD-mediated U747 methylation promotes efficient G748 methylation by methyltransferase RlmAII in 23S rRNA in Streptococcus pneumoniae; interplay between two rRNA methylations responsible for telithromycin susceptibility
Source: Nucleic Acids Res. 2015 Oct 10;43(18):8964–72. doi: 10.1093/nar/gkv609 (PMC4605293; doi:10.1093/nar/gkv609)
Supplement: SUPPLEMENTARY DATA [file supp_gkv609_nar-00714-r-2015-File008.pdf]

Supplementary Tables

Table S1 Bacterial strains used in this study

Strain	Relevant characteristics	Reference of source
Streptococcus pneumoniae		
TIGR4	Prototroph	Purchased from ATCC
ATCC49619	Drug sensitive	Purchased from ATCC
S1	TEL resistance clinical isolate	(10)
Sp224	TIGR4 harboring pTKY1041	This study
Sp274	Δ*tlrB*::*aad*(9) in S1	(10)
Sp303	Δ*tlrB*::*aad*(9) in TIGR4	(10)
Sp322	Sp303 harboring pTKY1111	This study
Sp331	Δ*SP_1901*::*aad*(9) in TIGR4	This study
Sp332	Δ*rlmCD*::*aad*(9) in TIGR4	This study
Sp338	Sp332 harboring pTKY1041	This study
Sp345	Δ*rlmCD*::*aad*(9) in S1	This study
Sp355	Sp345 harboring pTYK1111	This study
Sp360	Sp345 harboring pTKY1196	This study
Sp361	Sp332 harboring pTKY1196	This study
Sp369	Δ*rlmCD*::*erm*(B) in Sp303	This study
Sp370	Sp332 harboring pTKY1121	This study

Table S2 Plasmids used in this study

Plasmid	Relevant characteristics	Reference of source
pUC18	Cloning vector	Lab. collection
pLZ12-Km2	Shuttle vector	(17)
pAM225	Shuttle vector:pBR325 carrying Tn*917*	(18)
pGEX-6p-1	GST gene fusion vector	GE Healthcare
pTKY862	pLZ12-Km2 with Sp resistant cassette *aad*(9)	(17)
pTKY1041	pLZ12-Km2 with 1401 bp *erm*(B) fragment from S1	(10)
pTKY1109	pUC18 with 328 bp *tlrB* fragment	(10)
pTKY1110	pUC18 with disrupted *tlrB* fragment by insertion of an Sp resistant cassette *aad*(9)	(10)
pTKY1111	pLZ12-Km2 with 1065 bp *tlrB* fragment from S1	(10)
pTKY1121	pLZ12-Km2 with 1065 bp *tlrB* fragment from TIGR4	This study
pTKY1156	pUC18 with bp *SP_1901* fragment	This study
pTKY1157	pUC18 with 1240 bp *rlmCD* fragment	This study
pTKY1161	pUC18 with disrupted *SP_1901* fragment by insertion of an Sp resistant cassette *aad(9)*	This study
pTKY1174	pUC18 with disrupted *rlmCD* fragment by insertion of an Sp resistant cassette *aad*(9)	This study
pTKY1196	pLZ12-Km2 with 2112 bp *rlmCD* fragmant from S1	This study
pTKY1201	pGEX6p-1 with 864 bp *tlrB* fragment from TIGR4	This study
pTKY1205	pUC18 with disrupted *rlmCD* fragment by insertion of an Em resistant cassette *erm*(B).	This study

Supplementary Figures

Figure S1 Amino acid alignment of *E.coli* RlmC, *S. pneumoniae* SP_1029 and SP_1901, and *B. subtilis* YefA.

```
E.coli RlmC       1 ----------------------------------------------------------------------------MQ 2
Sp1029            1 -----MLKKNDIVEVEIVDLTHEGAGVAKVDGLVFFVENALPSEKILMRVLKVNKKIGFGKVEKYLVQSPHRNQDLDLAY 75
Sp1901            1 -------------------MGINGEGIGFYQKTLVFVPGALKGEDIYCQITSIRRNFVEAKLLKVNKKSKFRIVPSCTIY 61
B.subtilis YefA   1 MKMKPPVEKNEYYDVTFEDLTHEGAGVAKVQGFPIFVPNALPEEKAQIKVTRVKKGFAFGRLIELKEESPHRTDAPCPIY 80

E.coli RlmC       3 CALYDAGRCRSCQWIMQPIPEQLSAKTADLKNLLADFPVEEWCAPVSGPEQGFRNKAKMVVSGSVEKPLLGMLHRDG-TP 81
Sp1029           76 LRSGIADLGHLSYPEQLKFKTKQVKDSLYKIA--GIADVEVAETLGMEHPVKYRNKAQVPVRRVNGVLETGFFRKNSHNL 153
Sp1901           62 NECGGCQIMHLHYDKQLEFKTDLLHQALKKFAPAGYENYEIRPTIGMQEPKYYRAKLQFQTRKFKNQVKAGLYAQNSHYL 141
B.subtilis YefA  81 KQCGGCQLQHMTYEGQLLFKQKQVKDVLERIGKLDLSKVTVHPTLGMEDPWNYRNKAQVPVGEREGGLVAGFYQQRSHDI 160

E.coli RlmC      82 EDLCDCPLYPASFAPVFAALKPFIARAGLTPYNVARKRGELKYILLTESQSDGGMMLRFVLRSDTKLAQLRKALPWLHEQ 161
Sp1029          154 MPLEDFFIQDPVIDQVVVALRDLLRRFDLKPYDEKEQSGLIRNLVVRRGHYSGQIMVVLV-TTRPKVFRVDQLIEQVIKQ 232
Sp1901          142 VELKDCLVQDKETQVIANRLAELL-TYHQIPITDERKVLGVRTIMVRRARKTGQVQIIIV-TNR--QLNLTQLVKELVKD 217
B.subtilis YefA 161 IDMSACLIQQSKNDEAVQAVKDICANYGVKAYNEERHKGWLRHIMVRYGVVTGEMMIVFI-TRTSDFPHKAKIIEDITAQ 239

E.coli RlmC     162 LPQLKVITVNIQPVHMAIMEGETEIYLTEQQALAERFNDVPLWIRPQSFFQTNPAVASQLYATARDWVRQLPVKHMWDLF 241
Sp1029          233 FPEIVSVMQNINDQNTNAIFGKEWRTLYGQDYITDQMLGNDFQIAGPAFYQVNTEMAEKLYQTAIDFAELKKDDVIIDAY 312
Sp1901          218 FPEVVTVAVNTNTAKTSEIYGEKTEIIWGQESIQEGVLNYEFSLSPRAFYQLNPEQTEVLYSEAVKALDVDKEDHLIDAY 297
B.subtilis YefA 240 FPHVKSIVQNINPNKTNVIFGNETNVIWGEEYIYDLIGDVKFAISARSFYQVNPEQTKVLYDKALEYAELQGEETVIDAY 319

E.coli RlmC     242 CGVGGFGLHCATPDMQLTGIEIASEAIACAKQSAAELGLTRLQFQALDSTQFATA---QGDVPELVLVNPPRRGIGKPLC 318
Sp1029          313 SGIGTIGLSVAKHVKEVYGVELIPEAVENSQKNASLNKITNAHYVCDTAENAMKKWLKEGIQPTVILVDPPRKGLTESFI 392
Sp1901          298 CGVGTIGFAFAKKVKTLRGMDIIPEAIEDAKRNAKRMGFDNTHYEAGTAEEIIPRWYKEGYRADALIVDPPRTGLDDKLL 377
B.subtilis YefA 320 CGIGTISLFLAKQAKKVYGVEIVPEAIEDAKRNAELNGNTNAEFAVGEAETVIPKWYEEGITADTLVVDPPRKGCDEALL 399

E.coli RlmC     319 DYLSTMAPRFIIYSSCNAQTMAKDIRELP--GFRIERVQLFDMFPHTAHYEVLTLLVKQ--------------------- 375
Sp1029          393 KASAQTGADRIAYISCNVATMARDIKLYQELGYELKKVQPVDLFPQTHHVETVALLSKLDVDKHISVEIELDEMDLTSAE 472
Sp1901          378 DTILTYVPEKMVYISCNVSTLARDLVRLVEV-YDLHYIQSVDMFPHTARTEAVVKLIKKV-------------------- 436
B.subtilis YefA 400 RTIVEMKPKRVVYVSCNPGTLARDLRVLEDGGYVTREVQPVDMFPHTNHVECCVLIKLKE-------------------- 459

E.coli RlmC     375 -----------------------------------------------------------------------         375
Sp1029          473 SKATYAQIKEYVWNKFELKVSTLYIAQIKKKCGIELREHYNKSKKDKQIIPQCTPEKEEAIMDALRHFKMI         543
Sp1901          436 -----------------------------------------------------------------------         436
B.subtilis YefA 459 -----------------------------------------------------------------------         459
```

Figure S2 Level of m^1G748 in 23S rRNAs prepared from the parent strain TIGR4 or its derivatives.

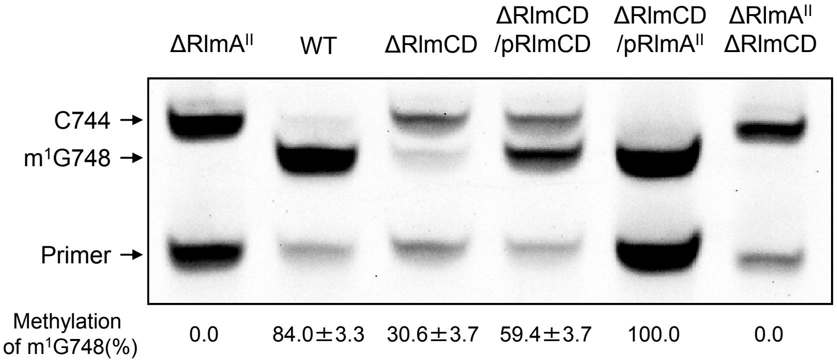

rRNAs prepared from strains TIGR4 (WT), Sp303 (ΔRlmA^{II}), Sp332 (ΔRlmCD), Sp360 (ΔRlmCD/pRlmCD), Sp369 (ΔRlmA^{II}ΔRlmCD) and Sp370 (ΔRlmCD/pRlmA^{II}) were sequenced by reverse transcriptase extension.

Figure S3 Time-course of m^1G748 formation in 23S rRNA catalyzed by 10nM $RlmA^{II}$.

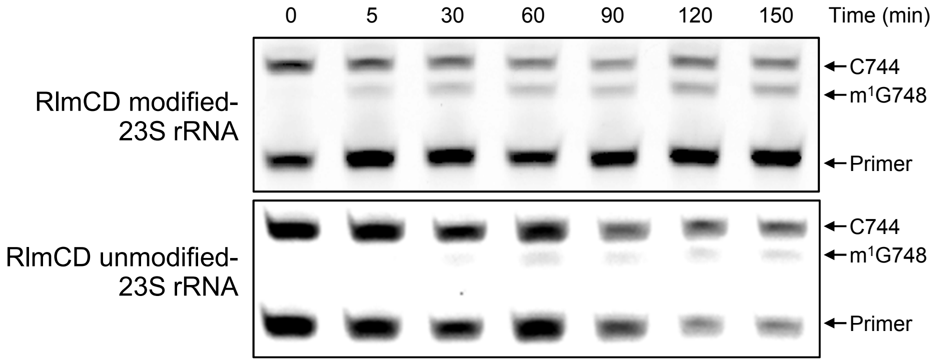

rRNAs (10nM) prepared from strains Sp303 (RlmCD modified-23S rRNA; upper panel) and Sp369 (RlmCD unmodified-23S rRNA; lower panel) were catalyzed by 10nM $RlmA^{II}$. The methylation was stopped using phenol solution at different times and the level of m^1G748 was analyzed by reverse transcriptase extension.
